# Supplementary material for: BRCA2-DSS1 interaction is dispensable for RAD51 recruitment at replication-induced and meiotic DNA double strand breaks
Source: Nat Commun. 2022 Apr 1;13:1751. doi: 10.1038/s41467-022-29409-y (PMC8975877; doi:10.1038/s41467-022-29409-y)
Supplement: Supplementary file 1 — Supplementary Information [file 41467_2022_29409_MOESM1_ESM.pdf]

SUPPLEMENTARY INFORMATION

Supplementary Figure 1: Generation of *Brca2*^{L2431P} knock-in mice.

a) Schematic representation of the targeting strategy to generate *Brca2*^{L2431P} knock-in allele by homologous recombination in mESC. A targeting vector containing 5.4kb 5′ homology and 4.5kb 3′ homology arms and a *loxP-PGK-neo-loxP* cassette for positive selection and *Thymidine Kinase (TK)* for negative selection was used to target the wild type locus (*Brca2*^{WT}). The 5’ homology arm contains CTG<CCA mutation in exon 15 that results in a leucine to proline substitution in codon 2431. Homologous recombination of the targeting vector resulted in the generation of *Brca2*$^{L2431P-Neo}$ allele. Mice heterozygous for *Brca2*$^{L2431P-Neo}$ allele were crossed with Cre expressing mice to delete the *Neo* cassette leaving behind a single *loxP* site in intron 15 to generate the *Brca2*^{L2431P} knock-in allele. Open boxes with numbers represent the exons with appropriate exon numbers and lines connecting the boxes represent the introns. Not drawn to scale. Relevant restriction sites are indicated, E: *Eco*RV; S: *Spe*I. b) We picked 72 G418 resistant colonies. Identified 19 to be correctly targeted by Southern analyses of genomic DNA digested with *Spe*I for 5′ end targeting and *Eco*RV for 3′ end targeting using probes shown in A (below the *Brca2*^{WT} locus). c) The mutation was confirmed by sequence analysis (3 clones). d) Schematic representation of the *Brca2* null allele (*Brca2*^{Ko}) showing deletion of 5′ region of exon 11, which is replaced with human *HPRT1* minigene.

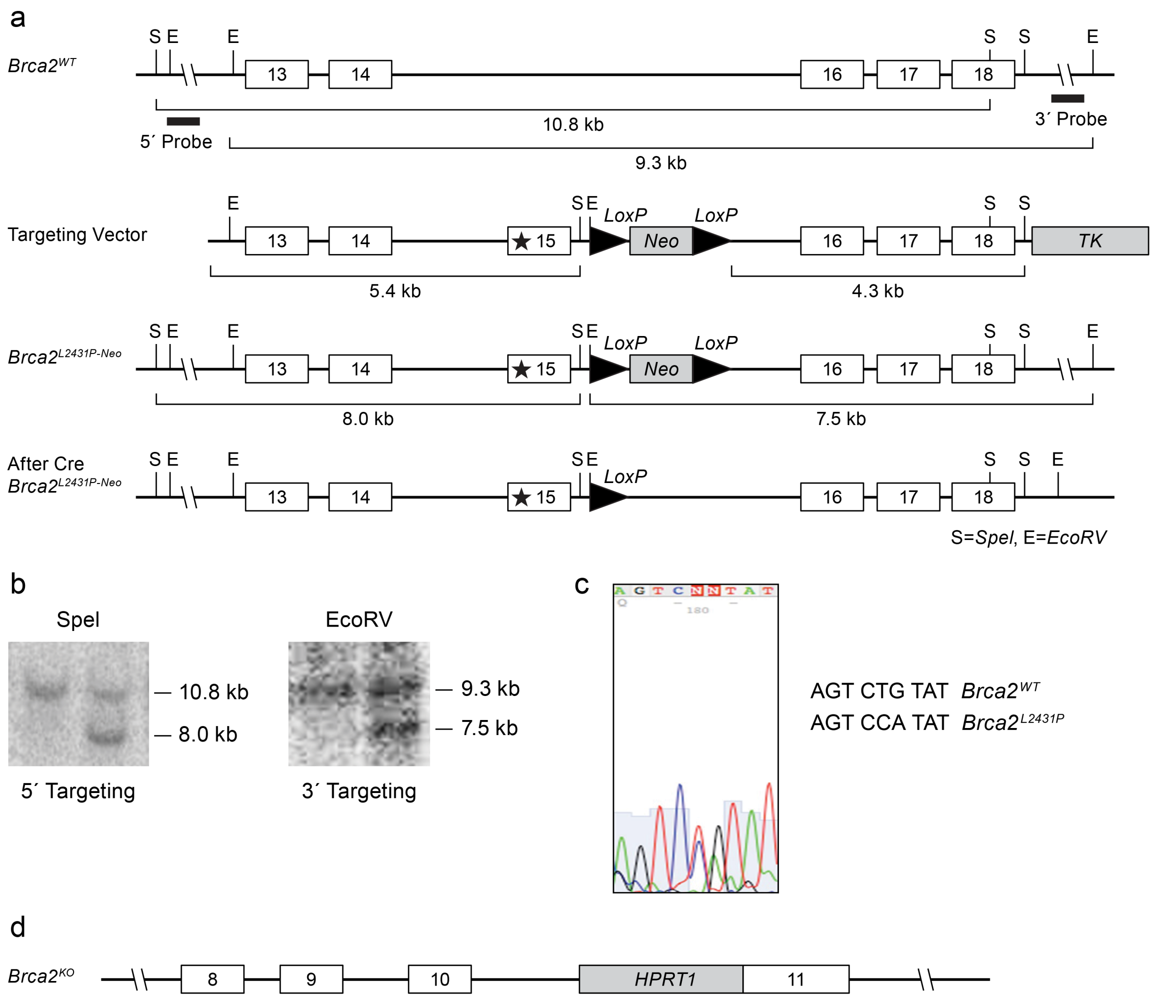
a
Brca2WT
S E E 13 14 16 17 18 S S E
10.8 kb
5´ Probe
9.3 kb
3´ Probe
Targeting Vector
E 13 14 15 S E LoxP Neo LoxP 16 17 18 S S TK
5.4 kb
4.3 kb
Brca2L2431P-Neo
S E E 13 14 15 S E LoxP Neo LoxP 16 17 18 S S E
8.0 kb
7.5 kb
After Cre
Brca2L2431P-Neo
S E E 13 14 15 S E LoxP 16 17 18 S S E
S=SpeI, E=EcoRV
b
SpeI
— 10.8 kb
— 8.0 kb
5´ Targeting
EcoRV
— 9.3 kb
— 7.5 kb
3´ Targeting
c
AGT CTG TAT Brca2WT
AGT CCA TAT Brca2L2431P
d
Brca2KO
8 9 10 HPRT1 11

Supplementary Figure 2: Whole mount of embryos and mammary gland .

a) Image of embryos at 16.5dpc obtained from an *LP/*+ intercross and from an *LP/*+ X *KO/*+ mating. b) Image of embryos at 18.5dpc obtained from an *LP/*+ intercross. c) H&E stained sagittal section of embryos at 16.5dpc of indicated genotypes. d) Representative images of carmine alum-stained mammary gland of 4-6 weeks old females of indicated genotype. The *LP/KO* mammary gland is smaller in size with reduced branching (scale bar=0.5cm). e) Quantification of number of terminal end buds observed in *WT* and *LP/KO* mammary glands. *LP/KO* shows significantly reduced number of terminal end buds as compared to WT (n=4 for WT and n=5 for LP/KO, Students two-tailed t-Test, error bar- SD of mean).

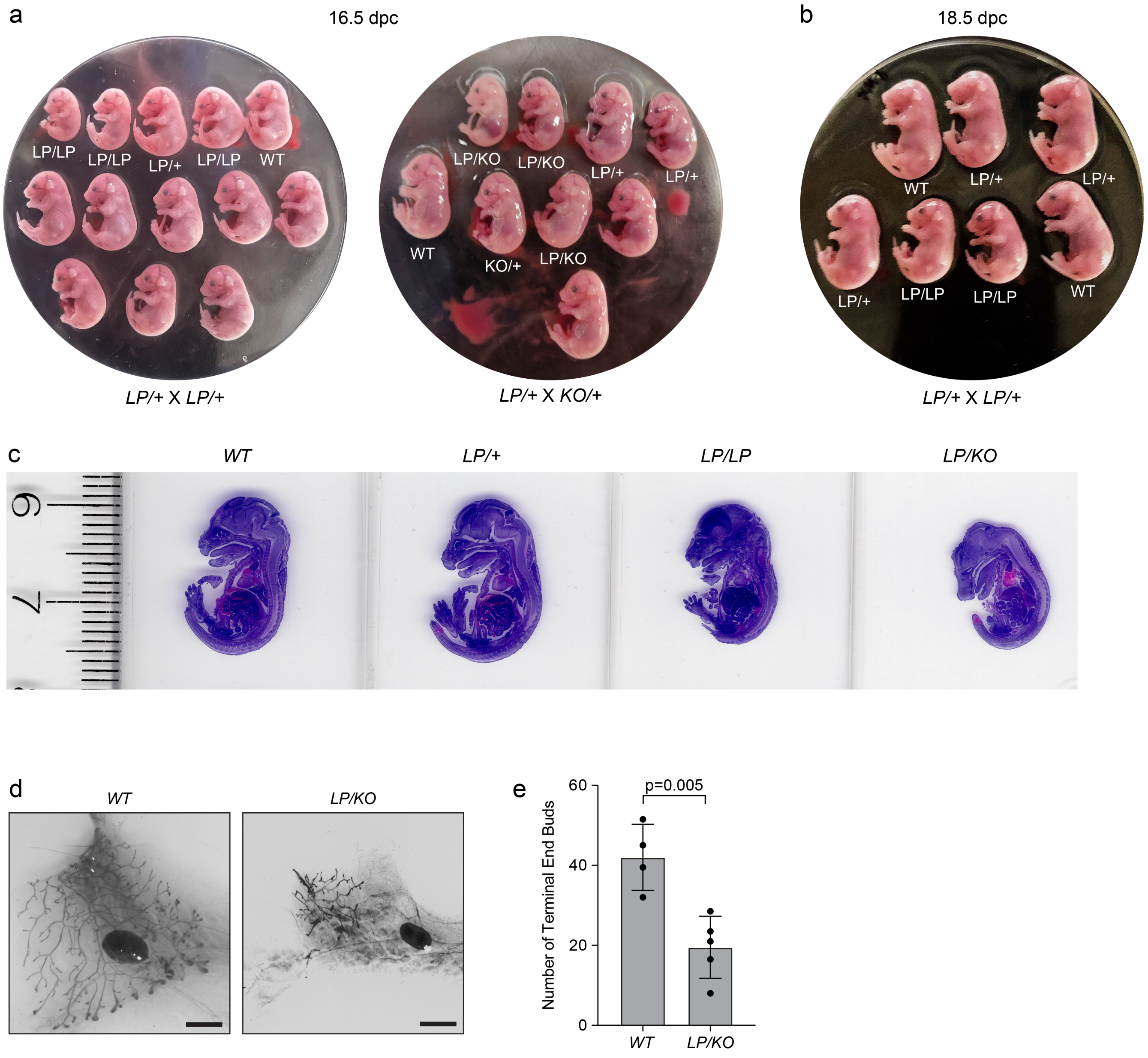
a
16.5 dpc
LP/LP
LP/LP
LP/+
LP/LP
WT
LP/+ X LP/+
LP/KO
LP/KO
LP/+
LP/+
WT
KO/+
LP/KO
LP/+ X KO/+
b
18.5 dpc
WT
LP/+
LP/+
LP/+
LP/LP
LP/LP
WT
LP/+ X LP/+
c
WT
LP/+
LP/LP
LP/KO
6
7
d
WT
LP/KO
e
p=0.005
Number of Terminal End Buds
60
40
20
0
WT
LP/KO

Supplementary Figure 3: Evaluation of genomic instability and protection of stalled replication forks in MEFs

a) Quantification of chromosomal aberrations observed in MEFs of indicated genotypes after 100nM MMC treatment shown in Figure 3c (n=3 MEFs , error bar- SD of mean). b) Scatter plot showing ratio of CldU:ldU (green:red) DNA fibers of MEFs of indicated genotypes after replication forks were stalled by 4mM HU treatment for 3hrs. *Brca1del11* MEFs were used as control for unprotected forks (n=200-300 fibers, error bar- SD of mean).

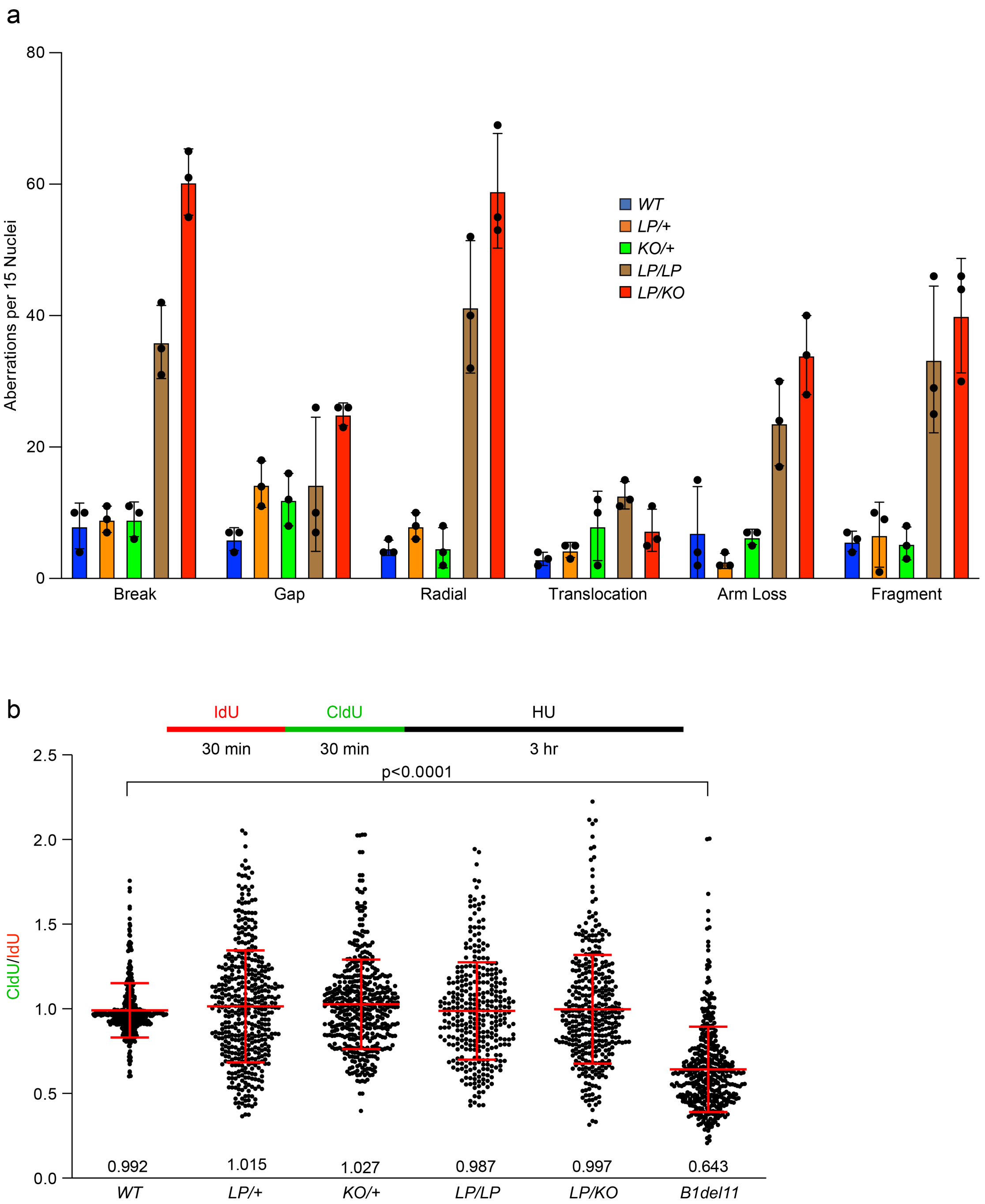
a
Aberrations per 15 Nuclei
80
60
40
20
0
WT
LP/+
KO/+
LP/LP
LP/KO
Break
Gap
Radial
Translocation
Arm Loss
Fragment
b
IdU
CldU
HU
30 min
30 min
3 hr
p<0.0001
CldU/IdU
2.5
2.0
1.5
1.0
0.5
0.0
0.992
1.015
1.027
0.987
0.997
0.643
WT
LP/+
KO/+
LP/LP
LP/KO
B1del11

Supplementary Figure 4: Histological analysis of testis and ovary of mutant and control mice.

a) H&E (upper panel) and TUNEL (lower panel) stained testes sections of 4-week old mice of indicated genotypes. H&E staining reveals normal microscopic testis morphology in all the genotypes. TUNEL staining suggests lack of abnormal apoptosis in the gonads of any genotype (n=3 mice per genotype, upper panel scale bar=50µm ,lower panel scale bar=200µm). b) H&E staining of 5-6 weeks old mice ovaries. Distinct Corpus lutea and developing follicles can be seen in each genotype (n=3 mice per genotype, scale bar=300µm). c) Representative immunofluorescence images of spermatocyte spreads from 4-week old mice of indicated genotypes in late pachytene/diplotene stages. The synapsed chromosomes are labeled with SCP3. γH2AX cloud can only be observed over the sex chromosomes in all the genotypes (scale bar=5µm). d) Quantification of the number of γH2AX foci observed per spermatocyte (error bar-SD of mean).

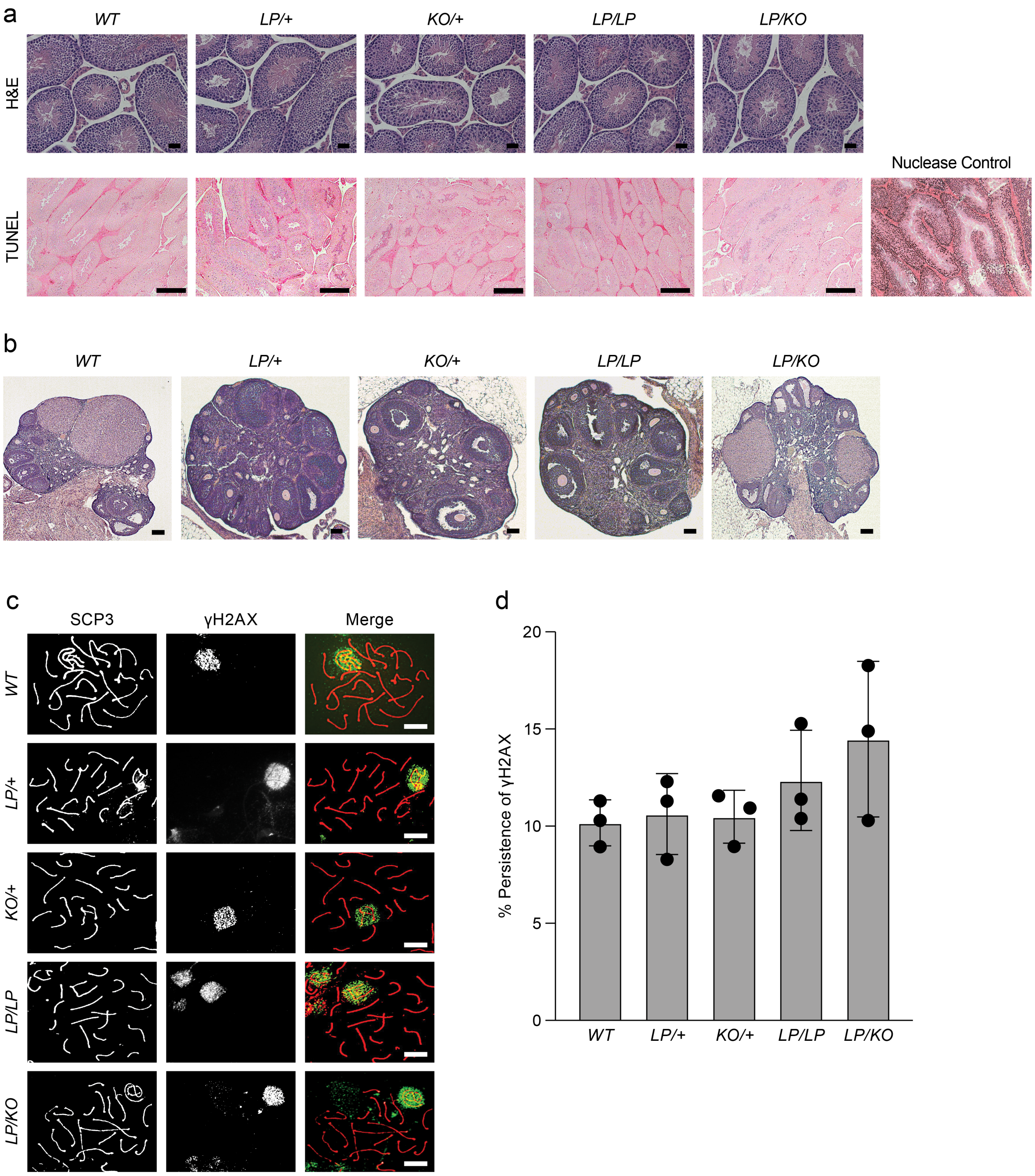
a
WT
LP/+
KO/+
LP/LP
LP/KO
H&E
TUNEL
Nuclease Control
b
WT
LP/+
KO/+
LP/LP
LP/KO
c
SCP3
γH2AX
Merge
WT
LP/+
KO/+
LP/LP
LP/KO
d
% Persistence of γH2AX
0
5
10
15
20
WT
LP/+
KO/+
LP/LP
LP/KO

Supplementary Figure 5: Effect of *Dss1* overexpression or heterozygosity on RAD51 foci formation.

a) Quantitative RT-PCR analyses to examine relative *Dss1* expression in MEFs transfected with vehicle or *Dss1* overexpression plasmid. b) Representative immunofluorescence images of MEFs of indicated genotypes for RAD51 loading after radiation induced DSBs with and without DSS1 overexpression. DSBs are marked by γH2AX and nuclei with DAPI (scale bar=5μm). c) Quantification of RAD51 foci per γH2AX positive nuclei in vehicle treated and DSS1 overexpressing cells. No significant change in the percentage RAD51 positive nuclei was observed in any genotype (n=3 biological replicates, ns-not significant, Students two-tailed t-Test, error bar-SD of mean). d) Representative immunofluorescence images of spermatocyte spreads from 4-week old mice of indicated genotypes on *Dss1*$^{+/-}$ background in early pachytene stages. The synapsed chromosomes are labeled with SCP3 (scale bar=5μm). e) Quantification of number of RAD51 foci observed per spermatocyte spread.

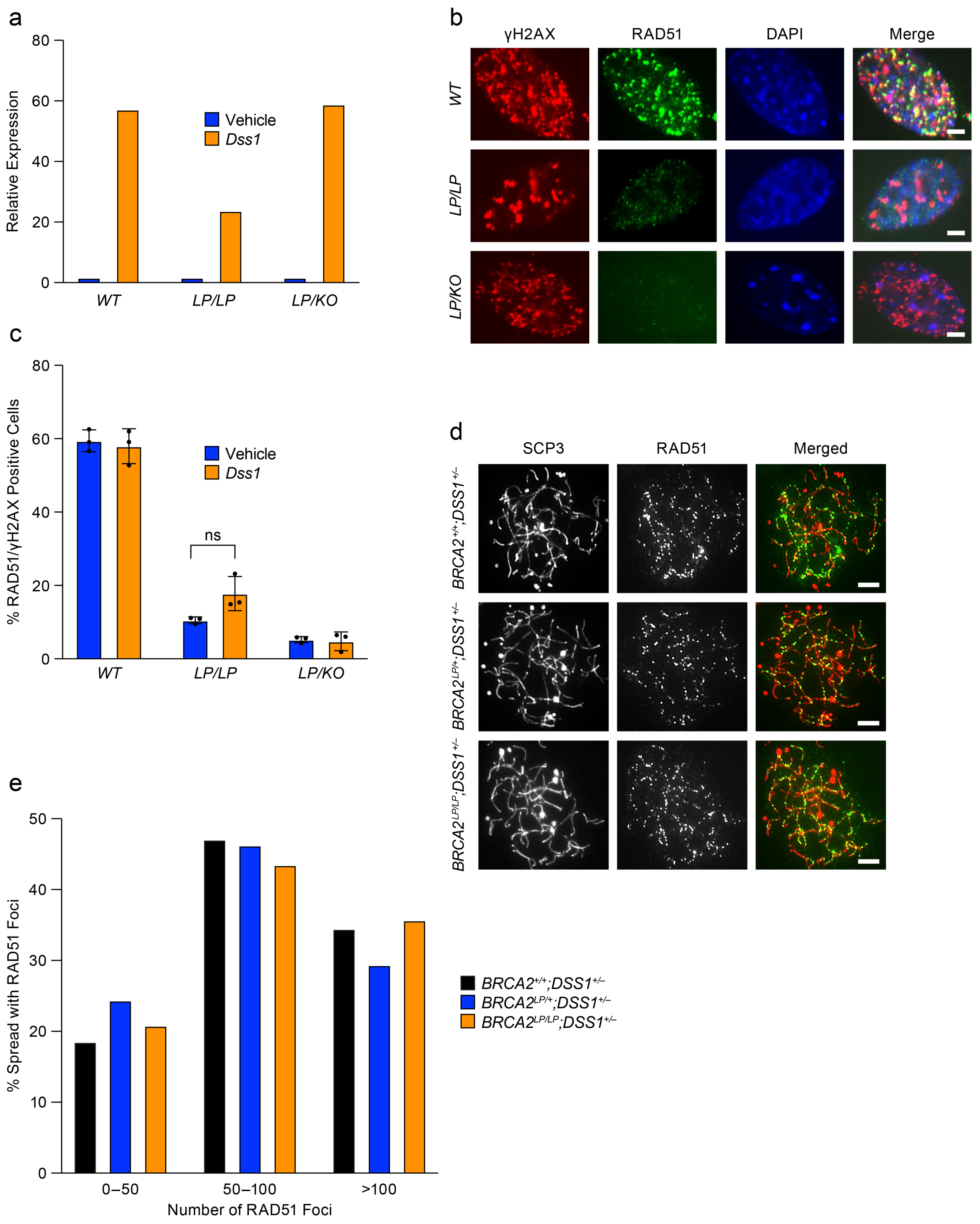

a
Relative Expression
80
60
40
20
0
Vehicle
Dss1
WT
LP/LP
LP/KO
b
γH2AX
RAD51
DAPI
Merge
WT
LP/LP
LP/KO
c
% RAD51/γH2AX Positive Cells
80
60
40
20
0
Vehicle
Dss1
ns
WT
LP/LP
LP/KO
d
SCP3
RAD51
Merged
$BRCA2^{+/+};DSS1^{+/–}$
$BRCA2^{LP/+};DSS1^{+/–}$
$BRCA2^{LP/LP};DSS1^{+/–}$
e
% Spread with RAD51 Foci
50
40
30
20
10
0
0–50
50–100
>100
Number of RAD51 Foci
$BRCA2^{+/+};DSS1^{+/–}$
$BRCA2^{LP/+};DSS1^{+/–}$
$BRCA2^{LP/LP};DSS1^{+/–}$

Supplementary Figure 6: Homologous recombination-mediated repair of radiation induced DSBs and Spo11-generated DSBs during meiosis I.

a) Schematic representation of different steps of homologous recombination occurring after radiation induced DSBs in somatic cells. DSS1 bound to BRCA2 displaces RPA32 from single stranded DNA (ssDNA) and facilitates loading of RAD51. RAD51 loaded ssDNA searches for homology resulting in strand invasion and D-loop formation. b) Schematic representation of different steps of homologous recombination occurring during meiosis I. The homologous chromosomes pair and condense together pre-leptotene and SPO11 mediated DSBs are generated. During pachytene, these DSBs get repaired by homologous recombination forming synaptonemal complex.

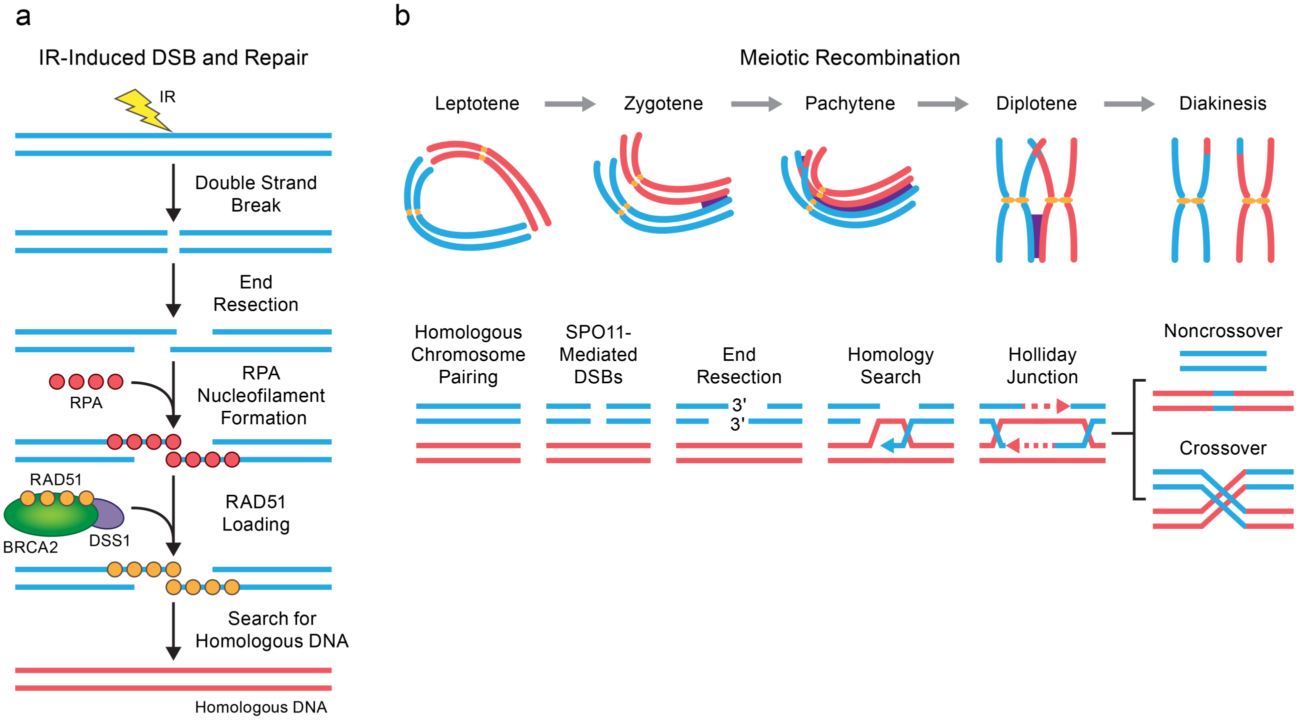
a
IR-Induced DSB and Repair
IR
Double Strand Break
End Resection
RPA
RPA Nucleofilament Formation
RAD51
BRCA2
DSS1
RAD51 Loading
Search for Homologous DNA
Homologous DNA
b
Meiotic Recombination
Leptotene
Zygotene
Pachytene
Diplotene
Diakinesis
Homologous Chromosome Pairing
SPO11-Mediated DSBs
End Resection
3'
3'
Homology Search
Holliday Junction
Noncrossover
Crossover

Supplementary Figure 7: EdU labeling and 53BP1 foci in MEFs in response to IR and Camptothecin treatment.

a) Representative immunofluorescence images of EdU labeling in WT MEF after indicated treatments. DSBs are marked with γH2AX and nuclei with DAPI (scale bar=10μm, experiment done with one WT MEF with different treatments). b) Representative immunofluorescence images of 53BP1 foci in MEFs of indicated genotypes exposed to IR or camptothecin. DSBs are marked with γ-H2AX and nuclei with DAPI (scale bar=5μm). c) Quantification of 53BP1 foci per γH2AX positive nuclei in untreated, IR treated and camptothecin treated cells. d) Chromosomal aberrations in MEFs of indicated genotype, untreated (top panels), 30nM camptothecin treated (middle panel) and 10nM olaparib (lower panel) treated. Chromosomal aberrations are marked with circles (scale bar=5μm). Quantification of these aberrations are shown in Fig. 5f.

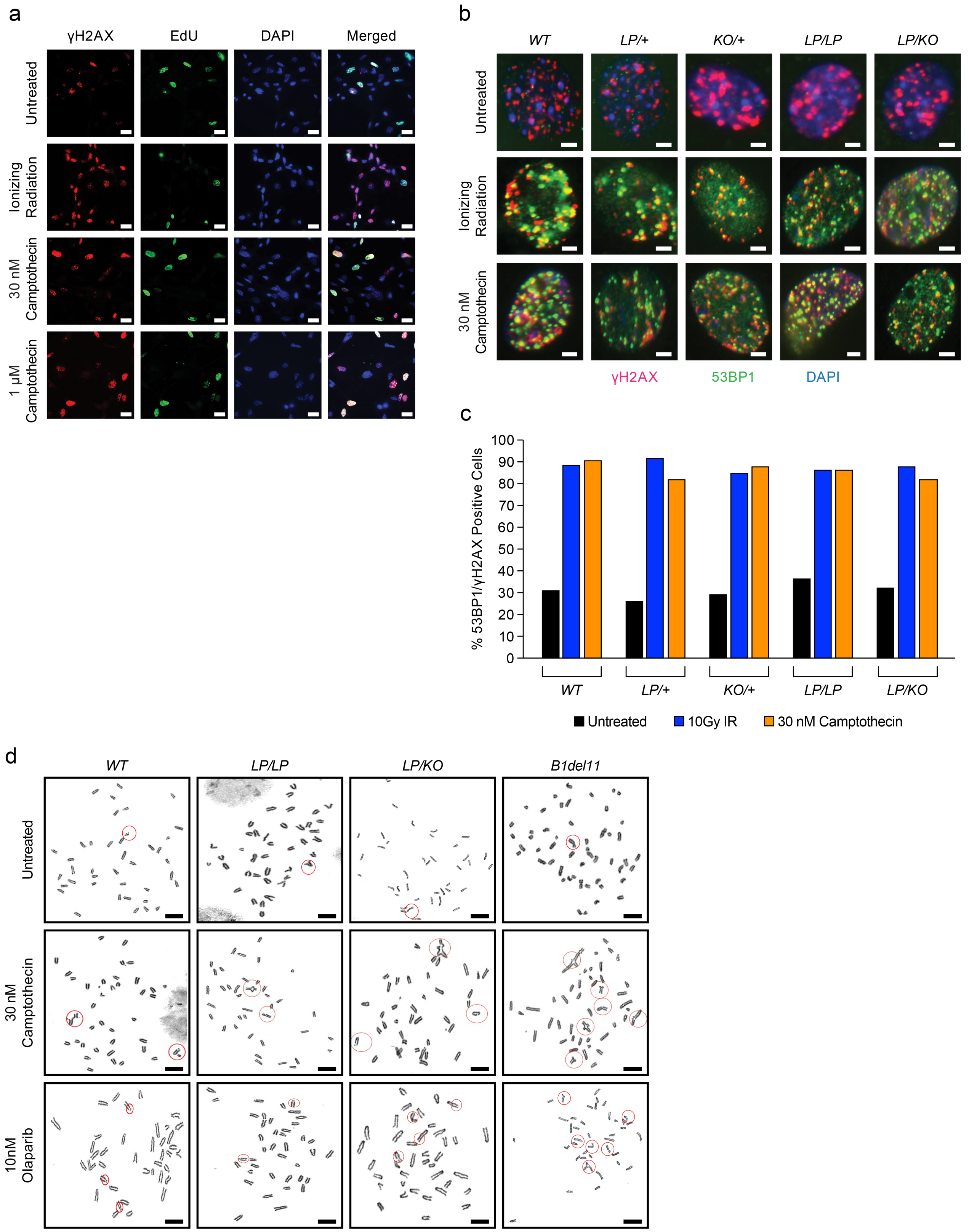

a
γH2AX
EdU
DAPI
Merged
Untreated
Ionizing Radiation
30 nM Camptothecin
1 μM Camptothecin
b
WT
LP/+
KO/+
LP/LP
LP/KO
Untreated
Ionizing Radiation
30 nM Camptothecin
γH2AX
53BP1
DAPI
c
% 53BP1/γH2AX Positive Cells
100
90
80
70
60
50
40
30
20
10
0
WT
LP/+
KO/+
LP/LP
LP/KO
Untreated
10Gy IR
30 nM Camptothecin
d
WT
LP/LP
LP/KO
B1del11
Untreated
30 nM Camptothecin
10nM Olaparib

Supplementary Figure 8: RAD51 foci at radiation and camptothecin induced DSBs in adult fibroblasts

a) Representative immunofluorescence images of RAD51 foci in adult fibroblasts of indicated genotypes exposed to 10Gy IR or 30nM camptothecin. DSBs are marked with γH2AX and nuclei with DAPI (scale bar=5μm). b) Quantification of RAD51 foci per γH2AX positive nuclei. Mutant MEFs are deficient in radiation induced RAD51 foci formation (n=3 biological replicates, Students two-tailed t-Test, error bar- SD of mean), but they show significant increase in RAD51 foci positive cells after camptothecin treatment (n=3, error bar- SD of mean).

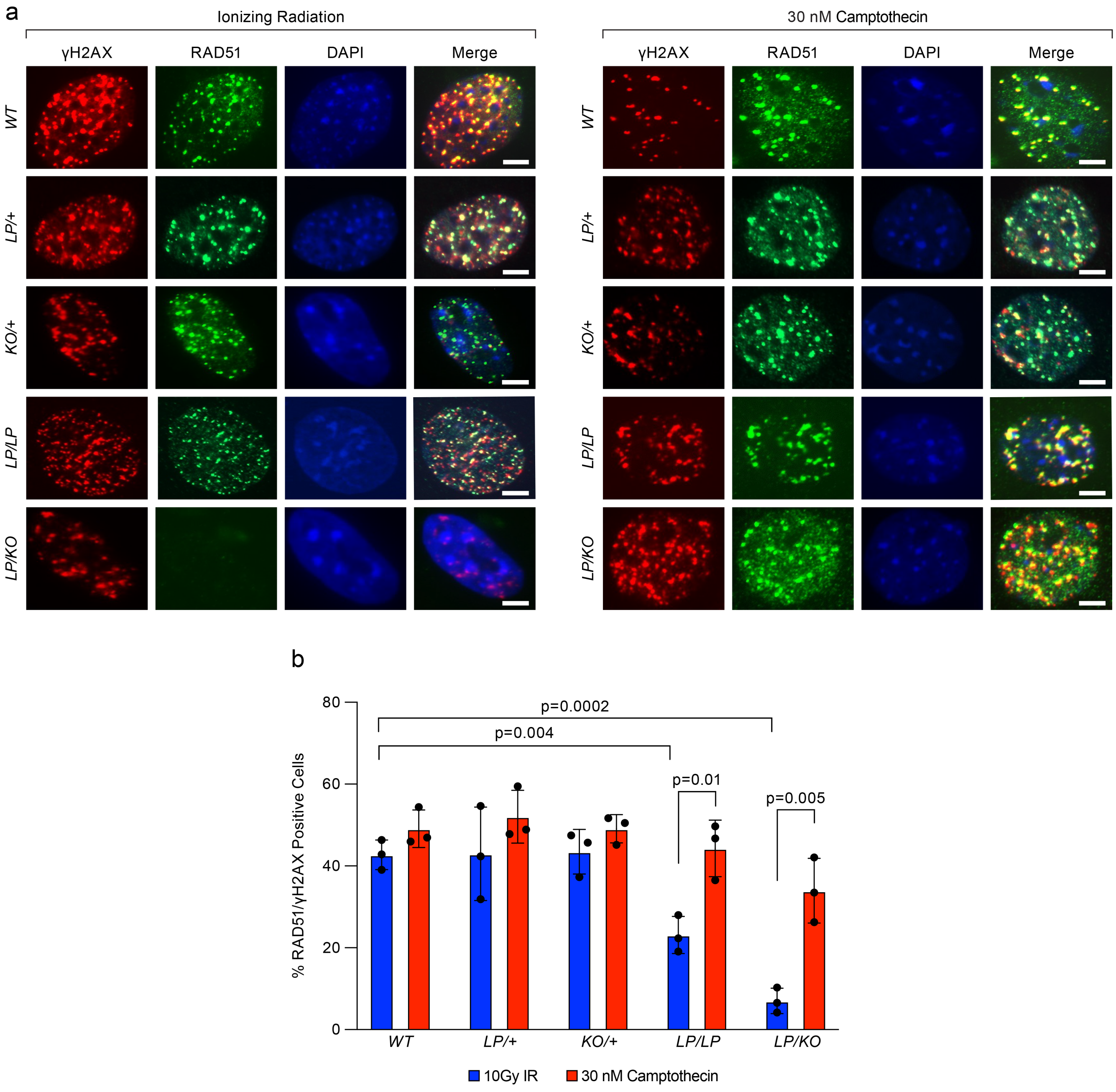
a
Ionizing Radiation
30 nM Camptothecin
γH2AX
RAD51
DAPI
Merge
WT
LP/+
KO/+
LP/LP
LP/KO
b
% RAD51/γH2AX Positive Cells
80
60
40
20
0
p=0.0002
p=0.004
p=0.01
p=0.005
WT
LP/+
KO/+
LP/LP
LP/KO
10Gy IR
30 nM Camptothecin

Supplementary Figure 9: CRISPR/Cas9-based homologous recombination assay.

a) Sequence of functional Blasticidin resistance gene (top) and the 29bp deletion that renders cells sensitive to blasticidin (bottom). Sequence of the donor DNA used to repair the 29bp deletion by HR is in the middle. PAM site in the blasticidin gene that was used to target by gRNA to generate DSB is marked by red box. b) Representative plates showing mESC colonies obtained in WT and BRCA2^{L2510P} complemented *Brca2$^{KO/KO}$* mESCs after transfection of Cas9 and gRNA expression vector and regular or biotinylated donor DNA. Colonies shown here were grown in media without blasticidin and the colony numbers were used as plating control with a dilution factor of 1:5000 to calculate HR frequency.

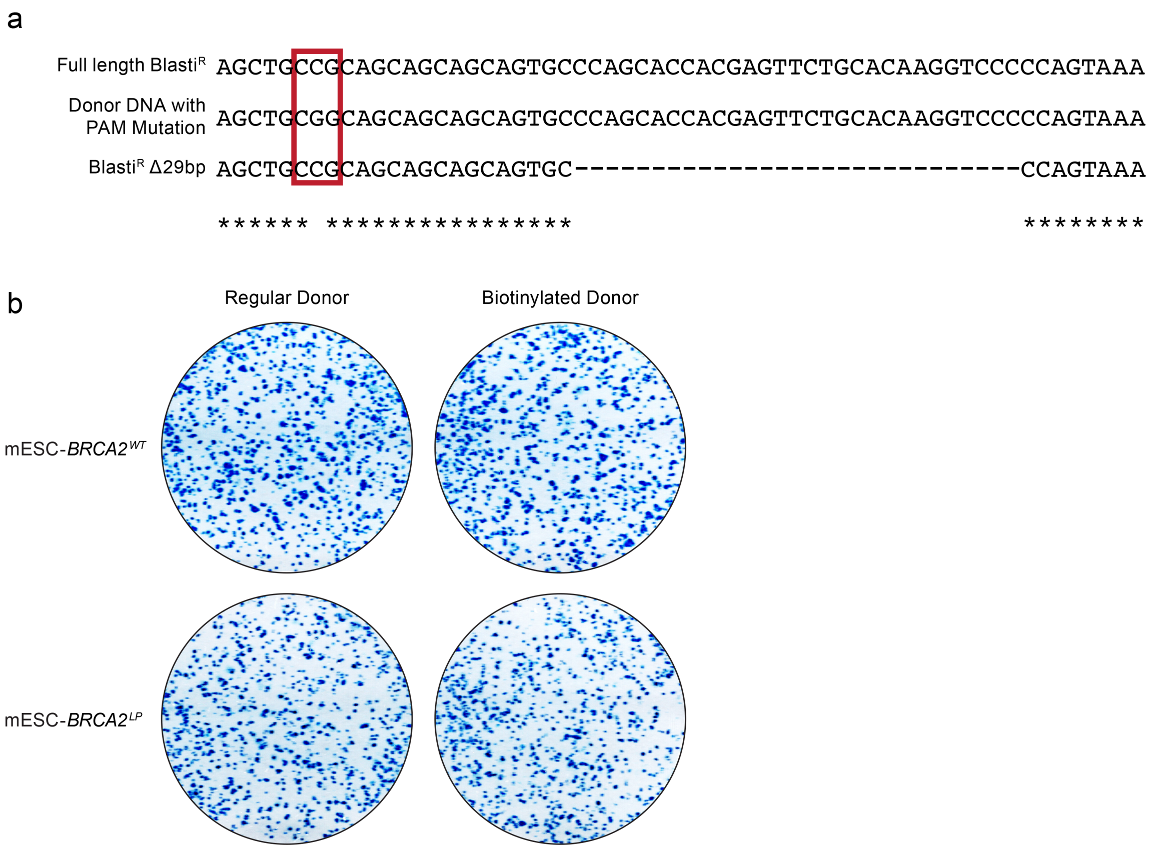

a
Full length Blasti^R AGCTGCCGCAGCAGCAGCAGTGCCCAGCACCACGAGTTCTGCACAAGGTCCCCCAGTAAA
Donor DNA with PAM Mutation AGCTGCGGCAGCAGCAGCAGTGCCCAGCACCACGAGTTCTGCACAAGGTCCCCCAGTAAA
Blasti^R Δ29bp AGCTGCCGCAGCAGCAGCAGTGC-----------------------------CCAGTAAA
****** **************** ********
b
Regular Donor
Biotinylated Donor
mESC-*BRCA2^{WT}*
mESC-*BRCA2^{LP}*

Supp Table 1: Average litter size of mating of mice of various genotypes

Mating (genotype of parents)	Average litter size (n)
LP/+ X +/+	7.44 (16)
LP/+ X LP/+	5.93 (20)
LP/LP X +/+	6.41 (12)
LP/KO X +/+	5.2 (5)
LP/LP X LP/LP	4.45 (24)
LP/LP X LP/KO	3.72 (18)
LP/KO X LP/KO	3.22 (23)

n represents number of litters

Supp Table 2: Observed and expected number of offspring of various genotypes obtained from *Brca2$^{LP/+}$;Dss1$^{+/-}$* X *Brca2$^{KO/+}$;Dss1$^{+/-}$* cross

Genotype	Observed	Expected	Mendelian Ratio	χ^2 p-value
Brca2$^{+/+}$;Dss1$^{+/+}$	15	9.625	1	1.76437E-17
Brca2$^{LP/+}$;Dss1$^{+/-}$	32	19.25	2	
Brca2$^{KO/+}$;Dss1$^{+/-}$	32	19.25	2	
Brca2$^{LP/+}$;Dss1$^{+/+}$	20	9.625	1	
Brca2$^{KO/+}$;Dss1$^{+/+}$	14	9.625	1	
Brca2$^{LP/KO}$;Dss1$^{+/+}$	6	9.625	1	
Brca2$^{LP/KO}$;Dss1$^{+/-}$	0	19.25	2	
Brca2$^{+/+}$;Dss1$^{+/-}$	35	19.25	2	
Brca2$^{LP/KO}$;Dss1$^{-/-}$	0	9.625	1	
Brca2$^{LP/+}$;Dss1$^{-/-}$	0	9.625	1	
Brca2$^{KO/+}$;Dss1$^{-/-}$	0	9.625	1	
Brca2$^{+/+}$;Dss1$^{-/-}$	0	9.625	1	

Supp Table 3: List of primers used in the study

Sequence	Amplicon size	Application
LP Fwd- ttagcctcacagcctctcctcgt<br>LP Rev- agagggccctatcaggtgtt	WT- 204bp<br>Mutant-320bp	Genotyping for l2510P locus
Hprt fwd- acagcatctaagaagttttgttctgtcctgg<br>E11 R- ctcaacagagtaggttctttgg	WT- no band<br>Mutant-450bp	Genotyping for *Brca2* Knockout allele
Delta neoS- ggctggtgtgtactgacaggat<br>Delta nesAS- ggcaaagcaggtgaacttgtgt	WT- 716bp<br>Mutant-242bp	Genotyping of DSS1 mutant animals
Dss1 rtf- cgacgagttcgaggagtttc<br>Dss1 rtr- ttctccagctcagcacgtaa	138bp	qPCR
Gapdh f-tgccccccatgtttgtgatg<br>Gapdh r-tgtggtcatgagcccttcc	151bp	qPCR
Blasti F- atcaacagcatccccatctc<br>Blasti R1- gcaattcacgaatcccaact	312bp	Cloning Blasticidin homologous donor DNA in TopoTA vector
Blasti F- atcaacagcatccccatctc<br>Blasti R2- tagccctcccacacataacc	WT- 339bp<br>mutant- 310bp	Genotyping Blasticidin resistant colonies
Blasti sgF- caccgactgggcactgctgctgctg<br>Blasti sgR- aaaccagcagcagcagtgcccagtc	20bp	Guide RNA for targeting mutant Blasticidin gene using CRISPR/Cas9
