# Supplementary material for: BRCA2-DSS1 interaction is dispensable for RAD51 recruitment at replication-induced and meiotic DNA double strand breaks
Source: Nat Commun. 2022 Apr 1;13:1751. doi: 10.1038/s41467-022-29409-y (PMC8975877; doi:10.1038/s41467-022-29409-y)
Supplement: Supplementary file 3 — Description of Additional Supplementary Information [file 41467_2022_29409_MOESM3_ESM.pdf]

Description of Additional Supplementary Information

SUPPLEMENTARY FIGURES

Supplementary Figure 1: Generation of *Brca2*^{L2431P} knock-in mice.

Supplementary Figure 2: Whole mount of embryos and mammary gland .

Supplementary Figure 3: Evaluation of genomic instability and protection of stalled replication forks in MEFs

Supplementary Figure 4: Histological analysis of testis and ovary of mutant and control mice.

Supplementary Figure 5: Effect of *Dss1* overexpression or heterozygosity on RAD51 foci formation.

Supplementary Figure 6: Homologous recombination-mediated repair of radiation induced DSBs and Spo11-generated DSBs during meiosis I.

Supplementary Figure 7: EdU labeling and 53BP1 foci in MEFs in response to IR and Camptothecin treatment

Supplementary Figure 8: RAD51 foci at radiation and camptothecin induced DSBs in adult fibroblasts

Supplementary Figure 9: CRISPR/Cas9-based homologous recombination assay.

SUPPLEMENTARY TABLES

Supp Table 1: Average litter size of mating of mice of various genotypes

Supp Table 2: Observed and expected number of offspring of various genotypes obtained from

Brca2$^{LP/+}$;*Dss1*$^{+/-}$ X *Brca2*$^{KO/+}$;*Dss1*$^{+/-}$ cross

Supp Table 3: List of primers used in the study
