# Supplementary material for: BRCA2-DSS1 interaction is dispensable for RAD51 recruitment at replication-induced and meiotic DNA double strand breaks
Source: Nat Commun. 2022 Apr 1;13:1751. doi: 10.1038/s41467-022-29409-y (PMC8975877; doi:10.1038/s41467-022-29409-y)
Supplement: Supplementary file 4 — Reporting Summary [file 41467_2022_29409_MOESM4_ESM.pdf]

nature portfolio

Corresponding author(s): Shyam K Sharan

Last updated by author(s): 03/09/2022

Reporting Summary

Nature Portfolio wishes to improve the reproducibility of the work that we publish. This form provides structure for consistency and transparency in reporting. For further information on Nature Portfolio policies, see our Editorial Policies and the Editorial Policy Checklist.

Statistics

For all statistical analyses, confirm that the following items are present in the figure legend, table legend, main text, or Methods section.

n/a	Confirmed	
☐	☒	The exact sample size (n) for each experimental group/condition, given as a discrete number and unit of measurement
☐	☒	A statement on whether measurements were taken from distinct samples or whether the same sample was measured repeatedly
☐	☒	The statistical test(s) used AND whether they are one- or two-sided<br>*Only common tests should be described solely by name; describe more complex techniques in the Methods section.*
☒	☐	A description of all covariates tested
☐	☒	A description of any assumptions or corrections, such as tests of normality and adjustment for multiple comparisons
☐	☒	A full description of the statistical parameters including central tendency (e.g. means) or other basic estimates (e.g. regression coefficient) AND variation (e.g. standard deviation) or associated estimates of uncertainty (e.g. confidence intervals)
☐	☒	For null hypothesis testing, the test statistic (e.g. F, t, r) with confidence intervals, effect sizes, degrees of freedom and P value noted<br>*Give P values as exact values whenever suitable.*
☒	☐	For Bayesian analysis, information on the choice of priors and Markov chain Monte Carlo settings
☒	☐	For hierarchical and complex designs, identification of the appropriate level for tests and full reporting of outcomes
☒	☐	Estimates of effect sizes (e.g. Cohen's d, Pearson's r), indicating how they were calculated

Our web collection on statistics for biologists contains articles on many of the points above.

Software and code

Policy information about availability of computer code

Data collection	*Provide a description of all commercial, open source and custom code used to collect the data in this study, specifying the version used OR state that no software was used.*
Data analysis	FlowJo, GraphPad prism9, Microsoft Excel, Snapgene viewer carl Zies (Zen blue 2.6)

For manuscripts utilizing custom algorithms or software that are central to the research but not yet described in published literature, software must be made available to editors and reviewers. We strongly encourage code deposition in a community repository (e.g. GitHub). See the Nature Portfolio guidelines for submitting code & software for further information.

Data

Policy information about availability of data

All manuscripts must include a data availability statement. This statement should provide the following information, where applicable:

- Accession codes, unique identifiers, or web links for publicly available datasets
- A description of any restrictions on data availability
- For clinical datasets or third party data, please ensure that the statement adheres to our policy

Only representative images shown in the figures. Summary of Histopathology data shown in Table 3, detailed report on each mouse not shown. available on request.

Field-specific reporting

Please select the one below that is the best fit for your research. If you are not sure, read the appropriate sections before making your selection.

☒ Life sciences ☐ Behavioural & social sciences ☐ Ecological, evolutionary & environmental sciences

For a reference copy of the document with all sections, see nature.com/documents/nr-reporting-summary-flat.pdf

Life sciences study design

All studies must disclose on these points even when the disclosure is negative.

Sample size	three or more biological replicates were used in all the mouse and MEF related experiments ('n' is mentioned in all the figures and/or legend). For ES cell based experiments three technical replicates were used as the cell line in use is verified line. For mouse experiments the animals were chosen randomly and n is mentioned in the figure legend
Data exclusions	No experimental data was excluded
Replication	All MEF or ES cell based experiments were conducted in triplicate, not result is excluded.
Randomization	Cell culture experiments were conducted by picking random MEFs. Groups were made on the basis of genotype.
Blinding	Blinding was done when the outcome was subjective and could be influenced by knowing the genotype. In all other cases no blinding was done.

Reporting for specific materials, systems and methods

We require information from authors about some types of materials, experimental systems and methods used in many studies. Here, indicate whether each material, system or method listed is relevant to your study. If you are not sure if a list item applies to your research, read the appropriate section before selecting a response.

Materials & experimental systems

n/a	Involved in the study	
☐	☒	Antibodies
☐	☒	Eukaryotic cell lines
☒	☐	Palaeontology and archaeology
☐	☒	Animals and other organisms
☒	☐	Human research participants
☒	☐	Clinical data
☒	☐	Dual use research of concern

Methods

n/a	Involved in the study	
☒	☐	ChIP-seq
☐	☒	Flow cytometry
☒	☐	MRI-based neuroimaging

Antibodies

Antibodies used	γH2AX (Millipore JBW301), RAD51 (Millipore PC130), 53BP1 (NovusBio NB100-304), RPA32 (Cell Signaling Technology 2208), mouse anti-SCP3 (santa cruz sc-74568), rabbit anti-SCP3 (Abcam ab15093), mouse anti-BrdU antibody (Becton Dickinson 347580), rat anti-BrdU antibody (Abcam ab6326), anti-biotin antibody (Bethyl-A150-109A)<br>Alexa-fluor anti-mouse 594 [Invitrogen A11005], anti-rabbit 488 [Invitrogen A11034], anti-mouse AlexaFluor488 (Invitrogen A21202), anti-rat AlexaFluor594 (Invitrogen A11007)
Validation	Mouse monoclonal anti phospho-histone H2A.X (Millipore JBW301)<br>Application: Immunofluorescence<br>Reference: Co-visualization of DNA damage and ion traversals in live mammalian cells using a fluorescent nuclear track detector. Kodaira, S; Konishi, T; Kobayashi, A; Maeda, T; Ahmad, TA; Yang, G; Akselrod, MS; Furusawa, Y; Uchihori, Y<br>Journal of radiation research 56 360-5 2015<br><br>Rabbit polyclonal RAD51 antibody (Millipore PC130)<br>Application: Immunofluorescence<br>Reference: CDK2 is required for proper homologous pairing, recombination and sex-body formation during male mouse meiosis In J. cell Science 2009. 122, 2149. by Viera et al.<br><br>Rabbit polyclonal 53BP1 Antibody (NovusBio NB100-304)<br>Application: Immunofluorescence<br>Reference: A transcription-based mechanism for oncogenic beta-catenin-induced lethality in BRCA1/2-deficient cells. Dagg RA, Zonderland G, Lombardi EP Et al. Nature communications Aug 13 2021 [PMID: 34389725]

Rat mAb RPA32 (Cell Signaling Technology 2208)
Application: Immunofluorescence
Reference: A meiosis-specific BRCA2 binding protein recruits recombinases to DNA double-strand breaks to ensure homologous recombination. Jingjing Zhang, et. al. Nat Commun. 2019; 10: 722.

mouse anti-SCP3 (sc-74568)
Application: Immunofluorescence
Reference: Antagonistic roles of ubiquitin ligase HEI10 and SUMO ligase RNF212 regulate meiotic recombination. Qiao, H. et al. 2014. Nature genetics. 46: 194-9.

Rabbit polyclonal anti-SCP3 (Abcam ab15093)
Application: Immunofluorescence
Reference- Fang K et al. Prediction and Validation of Mouse Meiosis-Essential Genes Based on Spermatogenesis Proteome Dynamics. Mol Cell Proteomics 20:100014 (2021).

mouse anti-BrdU antibody (Becton Dickinson 347580) Monoclonal (B44)
Application: Immunofluorescence
Reference- W Beisker, F Dolbeare, J W Gray. An improved immunocytochemical procedure for high-sensitivity detection of incorporated bromodeoxyuridine. Cytometry. 1987 Mar;8(2):235-9.

rat anti-BrdU antibody (Abcam ab6326) monoclonal [BU1/75 (ICR1)
Application: Immunofluorescence
Reference- Sebo ZL & Rodeheffer MS Testosterone metabolites differentially regulate obesogenesis and fat distribution. Mol Metab 44:101141 (2021).

anti-biotin antibody (Bethyl-A150-109A) Polyclonal
Application: Immunofluorescence
Reference- Escobar, Oksuz, Saldaña-Meyer et al. Active and Repressed Chromatin Domains Exhibit Distinct Nucleosome Segregation during DNA Replication. Cell (2019) 179 (4), 953-963

Alexa-fluor anti-mouse 594 [Invitrogen A11005]
Application: Immunofluorescence
Reference: Human-Induced Pluripotent Stem Cells Manufactured Using a Current Good Manufacturing Practice-Compliant Process Differentiate Into Clinically Relevant Cells From Three Germ Layers. Shafa M,Yang F,Fellner T,Rao MS,Baghbaderani BA. Front Med (Lausanne). 2018; 5: 69.

anti-rabbit 488 [Invitrogen A11034]
Application: Immunofluorescence
Reference: Human ectonucleotidase-expressing CD25high Th17 cells accumulate in breast cancer tumors and exert immunosuppressive functions. Thibaudin M,Chaix M,Boidot R,Végran F,Derangère V,Limagne E,Berger H,Ladoire S,Apetoh L,Ghiringhelli F. Oncoimmunology. 2016; 5(1): e1055444.

anti-mouse AlexaFluor488 (Invitrogen A21202)
Application: Immunofluorescence
Reference- Functional connectivity of intercalated nucleus with medial amygdala: A circuit relevant for chemosignal processing. IBRO Neurosci Rep (2022)

anti-rat AlexaFluor594 (Invitrogen A11007)
Application: Immunofluorescence
Reference- Chiang, HY., Lu, HH., Sudhakar, J.N. et al. IL-22 initiates an IL-18-dependent epithelial response circuit to enforce intestinal host defence. Nat Commun 13, 874 (2022)

Eukaryotic cell lines

Policy information about cell lines

Cell line source(s)	PL2F7 mouse embryonic stem cells. V6.4 mouse embryonic stem cells
Authentication	PL2F7 cell line is derived from AB2.2<br>Application- Stem cell research<br>Reference- Philipps Soriano, Charles Montgomery, Robert Geske, Allan Bradley, Targeted disruption of the c-src proto oncogene leads to osteopetrosis in mice, Cell, Volume 64, Issue 4, 1991, Pages 693-702, ISSN 0092-8674<br><br>PL2F7 mESc<br>Application- ES cell based drug screening<br>Reference: Mouse embryonic stem cell-based functional assay to evaluate mutations in BRCA2<br>In Nat Med. 2008 Aug;14(8):875-81. doi: 10.1038/nm.1719. Epub 2008 Jul 6. by Kuznetsov et al.<br><br>V6.4 mESc (C57BL/6J x 129S4/SvJae)F1<br>Application- Generating chimeric mice<br>Reference- You, Y., Bersgtram, R., Klemm, M. et al. Utility of C57BL/6J × 129/SvJae embryonic stem cells for generating chromosomal deletions: tolerance to γ radiation and microsatellite polymorphism. Mammalian Genome 9, 232–234 (1998).
Mycoplasma contamination	Cell line were routinely tested and are Mycoplasma free

Commonly misidentified lines (See ICLAC register)	No Commonly misidentified lines were used

Animals and other organisms

Policy information about studies involving animals; ARRIVE guidelines recommended for reporting animal research

Laboratory animals	C57Bl/6J Mus Musculus, males and females at all different age points (from E9.5 to 2 years of age) were used as mentioned in the manuscript.
Wild animals	No wild animals used
Field-collected samples	No field collected samples used
Ethics oversight	All mice were housed, bred and used in the study following the recommendations of the Guide for the Care and Use of Laboratory Animals (The National Academies Press; 8th edition). The study protocol was approved by the Animal Care and Usage Committee of NCI-Frederick (Animal Study# 18-471 and 21-471).

Note that full information on the approval of the study protocol must also be provided in the manuscript.

Flow Cytometry

Plots

Confirm that:

- [ ] The axis labels state the marker and fluorochrome used (e.g. CD4-FITC).
- [ ] The axis scales are clearly visible. Include numbers along axes only for bottom left plot of group (a 'group' is an analysis of identical markers).
- [ ] All plots are contour plots with outliers or pseudocolor plots.
- [ ] A numerical value for number of cells or percentage (with statistics) is provided.

Methodology

Sample preparation	10^5 cells (MEFs) of each genotype were plated in a 6 well plate in triplicate and viable cell counting using trypan blue was done at the indicated time points. MEFs of all genotypes were treated with Fxcycle PI/RNase solution (Invitrogen, F10797)
Instrument	BD FACS (BD LSRII)
Software	FlowJo
Cell population abundance	no sorting done
Gating strategy	Cell cycle phase gates (built in program of FlowJo)

- [ ] Tick this box to confirm that a figure exemplifying the gating strategy is provided in the Supplementary Information.
